# Twenty Years of the Health Insurance Portability and Accountability Act Safe Harbor Provision: Unsolved Challenges and Ways Forward

**DOI:** 10.2196/37756

**Published:** 2022-08-03

**Authors:** Brittany Krzyzanowski, Steven M Manson

**Affiliations:** 1 University of Minnesota Minneapolis, MN United States

**Keywords:** Health Insurance Portability and Accountability Act, HIPAA, data privacy, health, maps, safe harbor, visualization, patient privacy

## Abstract

The Health Insurance Portability and Accountability Act (HIPAA) was an important milestone in protecting the privacy of patient data; however, the HIPAA provisions specific to geographic data remain vague and hinder the ways in which epidemiologists and geographers use and share spatial health data. The literature on spatial health and select legal and official guidance documents present scholars with ambiguous guidelines that have led to the use and propagation of multiple interpretations of a single HIPAA safe harbor provision specific to geographic data. Misinterpretation of this standard has resulted in many entities sharing data at overly conservative levels, whereas others offer definitions of safe harbors that potentially put patient data at risk. To promote understanding of, and adherence to, the safe harbor rule, this paper reviews the HIPAA law from its creation to the present day, elucidating common misconceptions and presenting straightforward guidance to scholars. We focus on the 20,000-person population threshold and the 3-digit zip code stipulation of safe harbors, which are central to the confusion surrounding how patient location data can be shared. A comprehensive examination of these 2 stipulations, which integrates various expert perspectives and relevant studies, reveals how alternative methods for safe harbors can offer researchers better data and better data protection. Much has changed in the 20 years since the introduction of the safe harbor provision; however, it continues to be the primary source of guidance (and frustration) for researchers trying to share maps, leaving many waiting for these rules to be revised in accordance with the times.

## Introduction

### Background

When addressing many types of research problems, maps should generally be shared at a resolution that best portrays the reality of the underlying data. In terms of health and disease mapping, this realism often means desiring a fine-detailed visualization that helps make community-level public health interventions more effective. Geotechnology offers innovative ways of creating these fine-detailed maps and customizing them for the analysis and display of health data. However, at the same time, these data and tools can be dangerous when working with sensitive data, such as patient health records. In particular, scholars must be careful not to share maps that contain so much detail that individuals can be identified. To prevent the identification of patient records, in the United States, the Health Insurance Portability and Accountability Act (HIPAA) provides guidance on ways of deidentifying protected health information (PHI) before it is shared; however, HIPAA guidelines are difficult to apply to spatial data.

The HIPAA law poses several challenges to researchers seeking to use and share spatial data. First, many researchers find core elements of the *safe harbor provisions* of HIPAA (a set of conditions that define how data can be shared) ambiguous or difficult to understand, which is reflected in the disagreement and uncertainty in research and policy circles on how to meet the safe harbor standards. Second, playing it safe by taking a conservative approach to sharing maps to better meet the safe harbor standard—most often by releasing only highly aggregated maps or no maps at all—is a form of data loss that imposes potentially serious costs as it does not allow for the examination of local health distributions at reasonable resolutions for many common health problems. These 2 challenges lead to disagreement on how to follow privacy rules, and in fact, many scholars and policy makers have challenged these rules, saying that it is possible to share finer-grained mapped health data without jeopardizing patient privacy.

Addressing the twin challenges of the safe harbor provisions (ambiguity and data loss) requires an exploration of past and current understanding of how the provisions are enacted and identification of specific ways in which finer-scaled data may be legally and technically possible. The following section of this paper begins this exploration by examining the legal dimensions of the HIPAA law, from its creation to current practice. This section examines the events and concerns that fueled the motivations of those who helped write the safe harbor provisions, with a particular focus on answering the question of why zip codes and a population threshold of 20,000 were chosen as anchors for the safe harbors. The following section explores the first of the twin challenges—uncertainty—and establishes how some unintentional ambiguity in the law has led to different interpretations of HIPAA privacy provisions specific to geographic data in the public health literature. We focus on how this ambiguity has led to 2 common but different interpretations across a range of scholarships based on 3-digit and 5-digit zip codes and what this means for mapped data. The following section presents and explores data loss, the second of the twin challenges of the safe harbor provisions. The section builds on the previous ones to explore whether there is a middle ground between sufficiency and stringency, asking, in essence, if there are ways of minimizing risk under HIPAA while allowing for more useful maps. This paper concludes by presenting new approaches to the deidentification of patient data and discusses ways forward.

This paper advances our understanding, and potential use, of the safe harbor provision of HIPAA law, as applied to spatial data presented as maps. It is the first comprehensive overview of the long-standing and important conversations on this general topic. By untangling the law and reviewing its history and use, this paper offers avenues for finding safe and more useful ways of sharing mapped patient data. In addition, it seeks to spur a broader conversation on ways forward that necessarily expand and improve shared understanding of privacy regulations to encourage researchers to investigate alternative strategies.

## HIPAA Privacy Act: Zip Codes and the 20,000-Person Population Threshold

### Overview

To better understand the safe harbor provision and what it asks of researchers, it is best to first understand its origin. Examining HIPAA in terms of its history and evolution sheds light on how to approach the sharing of geographic information under the safe harbor standard. We asked two related questions: (1) why do zip codes hold such sway over defining the safe harbor rule, and (2) why is a threshold of 20,000 people used to define privacy? Answering these questions clarifies some of the key ambiguities in HIPAA safe harbors and provides insight into why there is so much seeming disagreement within and across research domains. The following section provides a brief overview of HIPAA privacy law before diving into the history of the safe harbor provision to provide insights into the 2 key ambiguities (the use of zip codes and the population threshold).

### The Safe Harbor Provision

To protect patient privacy, HIPAA limits the ways in which patient data can be shared. Patient data are considered PHI that needs to be kept secure as they include private medical information along with identifying information such as names, birth dates, addresses, and social security numbers. Address data, in particular, are considered extremely sensitive as they (along with other location data such as longitude and latitude) may be used to pinpoint the residence of an individual. This degree of locational specificity substantially increases the likelihood of identification, if not fully guaranteeing identification in the case of single-occupant residences. For this reason, patient locations need to be masked in accordance with HIPAA privacy law.

Two standards are specified under the HIPAA rule for deidentifying patient data—the safe harbor standard and expert determination—but the former is the de facto standard [1]. Expert determination—also termed as the statistical standard—is the process by which an investigator masks their data and has a third-party expert determine whether the applied location masking strategy provides a low probability of identification [1]. Expert determination is not frequently used in large part as it is ambiguous and requires unspecified documentation, in addition to placing a great deal of pressure on the third-party expert who is charged with certifying HIPAA compliance. This leaves the safe harbor standard as the most commonly relied upon practice for deidentifying patient data [2]. Its immediate appeal, and the primary reason for broader acceptance than expert determination, is that it offers ostensibly clear guidance. The safe harbor standard is the focus of the remainder of this paper.

In essence, the safe harbor method protects patient data by simply removing 18 types of identifiers (Textbox 1). Many of these elements are straightforward to comprehend and implement, such as not including names, birth dates, and social security numbers. Some of the other elements pose their own challenges in an age of surveillance, such as biometric markers, including vehicle license plates and facial imagery. However, our focus is section 2 of the safe harbor relating to the patient’s location, which is especially relevant to mapping and, not surprisingly, the primary source of confusion in applying the safe harbor rule to mapping. The location provision of the safe harbor rule requires a minimum population of at least 20,000 people to be contained within each aggregated geographical unit, and the rule further requires that the only permissible geography (smaller than the state) be a form of zip code.

Ambiguity arises when the type of zip code is not specified. Although it seems fairly clear from Textbox 1 that the rule intends for investigators to rely on the use of 3-digit zip codes (compared with 5-digit zip codes), not all who read this stipulation see it that way. There are many reasons for this, including various misleading representations of the rule found in legal web-based documentation and in the literature on public health and disease mapping [3-11]. The following section explores how zip codes have come to play a key role in the safe harbor rule.

**The key elements of the safe harbor provision.**

The following identifiers of the individual or of relatives, employers, or household members of the individual, are removed:

1. Names
2. All geographic subdivisions smaller than a state, including street address, city, county, precinct, zip code, and their equivalent geocodes, except for the initial 3 digits of the zip code if, according to the current publicly available data from the Bureau of the Census, the geographic unit formed by combining all zip codes with the same 3 initial digits contains >20,000 people, and the initial 3 digits of a zip code for all such geographic units containing ≤20,000 is changed to 000
3. All elements of dates (except year) for dates that are directly related to an individual, including birth date, admission date, discharge date, death date, and all ages >89 years, and all elements of dates (including year) indicative of such age, except that such ages and elements may be aggregated into a single category of the age of ≥90 years
4. Telephone numbers
5. Vehicle identifiers and serial numbers, including license plate numbers
6. Fax numbers
7. Device identifiers and serial numbers
8. Email addresses
9. Web Universal Resource Locators (URLs)
10. Social security numbers
11. IP addresses
12. Medical record numbers
13. Biometric identifiers, including finger and voice prints
14. Health plan beneficiary numbers
15. Full-face photographs and any comparable images
16. Account numbers
17. Any other unique identifying number, characteristic, or code, except as permitted by paragraph c of this section (paragraph c is presented in the section “Re-identification”)
18. Certificate and license numbers

### Why Zip Codes?

If we were to remove zip codes from the safe harbor provision, there would be no ambiguity in terms of its interpretation as the rule would simply focus on the threshold of 20,000 people to define whether an arbitrary geographical unit is sufficient. Hence, why are zip codes still written into the law? To answer this, we need to start at the very beginning and understand how the political, social, and technological milieu of the early and mid-1990s shaped some core principles and guidelines. Zip codes were originally not included in the rule; however, this quickly changed as a result of a mix of happenstance and deliberation. The following paragraphs provide insight into the series of events that led to the HIPAA safe harbor provision that we understand today, beginning with the proposed bill.

Before HIPAA was law, it was a bill, specifically bill *H.R. 3103* of the 104th Congress from 1995 to 1996. This bill was introduced in the spring of 1996 as part of an initial attempt at health care reform by the Clinton administration. The overarching focus of H.R. 3103 was to improve access to health care and address fraud, waste, and abuse in health insurance and health care delivery; however, it also—quite briefly—mentions a specific interest in the protection of patient data (section 1177 of H.R. 3103, 1996). In a single paragraph, the bill addresses the wrongful disclosure of individually identifiable health information, in large part, as it relates to insurance fraud and abuse:

A person who knowingly and in violation of this part uses or causes to be used a unique health identifier; obtains individually identifiable health information relating to an individual; or discloses individually identifiable health information to another person, shall...be fined not more than $50,000, imprisoned not more than 1 year, or both; if the offense is committed under false pretenses, be fined not more than $100,000, imprisoned not more than 5 years, or both; and if the offense is committed with intent to sell, transfer, or use individually identifiable health information for commercial advantage, personal gain, or malicious harm, fined not more than $250,000, imprisoned not more than 10 years, or both.

Section 1177. Wrongful disclosure of individually identifiable health information

This bill was the first step toward the development of a series of protections that would eventually become the HIPAA privacy law that we know today. However, much changed during the journey from the bill’s initial proposal to the passage of the final law and attendant guidelines, especially in terms of modifications made to the data privacy and deidentification standards. Early renditions of HIPAA provided very little guidance on how to define deidentified health information. Mass computerization of individual health information had only just begun, with electronic health records making their first appearance in 1992 [12]. In the mid-1990s, with the rise of the internet and home computers, threats to data privacy elicited much fear among the American public [13]. Despite these concerns, when the bill went to Congress in the summer of 1996, the disclosure of identifiable health information was not documented as a part of the discussion on the congressional record [14].

A year after its introduction, Sweeney [15], a computer scientist working at the Massachusetts Institute of Technology, purchased a voter registration list for Cambridge, Massachusetts, United States, and cross-referenced it with a “de-identified” (meaning the names were missing but other information such as birth date remained) Massachusetts Group Insurance hospitalization data set that was provided to researchers. Sweeney [15] determined that by using birth date, gender, and a 5-digit zip code, she could match a patient’s medical records with their name on the voter registration list. This meant that for only US $20 (the cost of the voter registration list), Sweeney [15] could *potentially* identify (by name) some of the registered voters and their medical records, which included sensitive information such as diagnoses, procedures, and medications. With this knowledge, Sweeney [15] famously mailed the governor of Massachusetts his own medical records. This event fueled anxiety about the potential misuse of patient information and put data protection at the forefront of many conversations on privacy reform. The study by Sweeney [15] was central to the next chapter of the story of HIPAA’s evolution, the 1999 Notice of Proposed Rulemaking (NPRM) [16,17].

In response to the work by Sweeney [15], the 1999 NPRM proposed a stringent definition of deidentified health information. Of particular interest to this paper is how the NPRM defined the smallest unit of allowable geography as the state. All other geographic identifiers would be removed, meaning that street addresses, cities, counties, and both 3- and 5-digit zips were not permissible. This state-level geographic standard was too restrictive for any researcher interested in studying the geographic variation in health and disease, such as geographers and epidemiologists. Under such rules, researchers are only able to publish maps at the state level (usually at the national level). For most scholars, this limit meant that only statistical point estimates (such as regression output) could be published under the safe harbor rule.

Fortunately, for researchers, feedback from the 1999 NPRM’s call for public comments pushed the Department of Health and Human Services (HHS) to allow slightly more geographic information to be shared as deidentified information. The safe harbor standard’s 3-digit zip code rule made its first appearance on a federal record [18]. The rule states the following:

In the safe harbor, we explicitly allow...some geographic location information to be included in the deidentified information, but...zip codes must be removed or aggregated (in the form of most three-digit zip codes) to include at least 20,000 people.

Compared with the 1999 NPRM guidelines, this safe harbor standard was much less stringent but still meant to withstand a population-level identification attack of the sort developed by Sweeney [15], which required 5-digit zip codes.

This simple 3-digit zip code rule became more complicated in the decade after HIPAA was promulgated. The initial formulation seemed clear (3-digit zip codes were the intended level of aggregation); however, subsequent modifications to HIPAA introduced ambiguity. Changes to the final rule in 2002 left out the key clause that made it clear that 3-digit zip codes would be the *only* permissible form of aggregation (other than the state level) [19]. This contributed to the ever-growing ambiguity regarding the provision of geographic deidentification, and along with other nebulous aspects of the law, many researchers found it difficult to navigate HIPAA. Therefore, with the passage of the Health Information Technology for Economic and Clinical Health Act in 2009, the HHS was required “to issue guidance on methods for de-identification of PHI as designated in HIPAA’s Privacy Rule.” In response, the US Office of Civil Rights (OCR) held a workshop in 2010 to provide guidance on strategies for the deidentification of PHI. OCR used input from panelists, including Sweeney and Barth-Jones (noted later in this paper), and workshop attendees to develop a lengthy guidance document [1]. This comprehensive document is helpful in that it provides a more detailed description of the safe harbor rule; however, unfortunately, it still contained the same ambiguous phrasing (regarding zip codes) found in the modifications of the written law. To make matters worse, the landing page for the workshop on HIPAA’s deidentification standard (which features a link to the guidance document page) uses the term *geocodes* rather than zip codes (Textbox 2 provides the full phrasing) when referring to aggregating geographic data, which could easily lead readers to believe that any unit (not only zip codes) could be used for aggregation. These ambiguities, alongside inconsistencies in use and opinion found throughout the literature (explored below in section *Twin Challenge 1: Ambiguity*) about core HIPAA documents [1,19], may have contributed to the widespread confusion that continues today.

**The various ways investigators interpret the geographic location stipulation of the Health Insurance Portability and Accountability Act (HIPAA) safe harbor rule.**

**Paper, author, and interpretation**

- Confidentiality risks in fine scale aggregations of health data (Curtis et al [6])
  “Unfortunately there are few guidelines with regards the release of aggregated data. A commonly discussed threshold between researchers is that health data should only be visualized for ZIP codes with a base population of no less than 20,000.”
- Reidentification risks in HIPAA safe harbor data: a study of data from one environmental health study (Sweeney et al [10,20])
  “[T]he provision requires removing explicit identifiers (such as name, address and other personally identifiable information), reporting dates in years, and reducing some or all digits of a postal (or ZIP) code.”
- Workshop on the HIPAA privacy rule’s deidentification standard (US Office of Civil Rights [11])
  “[The Safe Harbor approach] permits a covered entity to consider data to be de-identified if it removes 18 types of identifiers (eg, names, dates, and geocodes on populations with less than 20,000 inhabitants) and has no actual knowledge that the remaining information could be used to identify an individual, either alone or in combination with other information.”
- Conforming to HIPAA regulations and compilation of research data (Clause et al [3])
  “Implementation of these methods can be somewhat difficult for the clinical researcher for data sets of less than 20,000 records (as determined by collapsing populated geographic codes representing sparse populations).”
- From healthy start to hurricane Katrina: using GIS to eliminate disparities in perinatal health (Curtis [4])
  “The error of recording ‘70808’ rather than ‘70806’ in Baton Rouge would involve considerable changes in social, economic, and racial contexts. This is a problem if data are only available by zip code, which unfortunately is still too common in terms of releasing data for GIS analysis.”
  “Although there are HIPAA regulations regarding the display of aggregate data on choropleth maps, these guidelines are generally considered too restrictive for useful cartography (only zip codes with more than 20 000 can be visualized).”
- A linear programming model for preserving privacy when disclosing patient spatial information for secondary purposes (Jung and El Emam [7])
  “A prevailing method to create de-identified data sets is to aggregate pre-defined areas, such as ZIP codes or counties, into a new area.”
  “Yet, the first three digits of a ZIP code may be included, provided that at least 20,000 people share the same first three digits.”
- The challenges of creating a gold standard for deidentification research (Browne et al [8])
  “[The guidelines of the Privacy Rule] say that units smaller than a state should be redacted, although Baltimore has a population of well over 20,000, the size limit for Zip-Codes. D.C. was considered a state for this purpose.”
- Challenges and insights in using HIPAA privacy rule for clinical text annotation (Kayaalp et al [9])
  “The Privacy Rule states that information about all geographic subdivisions smaller than state, except the first two digits of the zip code, must be de-identified. The third digit of the zip code can be left intact, only if the size of the population in the area of the censored two digits is greater than 20,000 according to the most recent census data.”
- Broken promise of privacy: responding to the surprising failure of anonymization (Ohm [5])
  “Id. § 164.514(b)(2)(B) (allowing only two digits for ZIP codes with 20,000 or fewer residents).”

### Why 20,000 People?

Part of the ambiguity surrounding the use of zip codes is tied to the 20,000-person threshold in defining safe harbor rules. The decision to allow substate-level geographies, specifically zip codes, is partially tied to research on the role of the population size in protecting privacy. In simple terms, by increasing the number of people reported within a given region, the chances of successfully matching an individual in that region to their health records decreases. This is because the odds of a unique combination of identifying characteristics occurring in a population decline as the number of people in a data set increases.

How did the HHS determine that 20,000 was the appropriate population threshold? To answer this, we must look to the proposed final rule [18] as there is little to no discussion of this determination within the literature or on the HHS support and guidance webpages. In the final rule, the HHS points to the precedent of how the Bureau of the Census “shares geographical units only if they contain populations of at least 100,000 people” [20]. This standard is conservative, and thus, the HHS turned to other sources so that they might be able to drop the threshold lower.

Specifically, the HHS drew on 2 simulation studies, one by Greenberg and Voshell [21] and the second by Horm [22]. These studies explored how the proportion of unique records within a data set can be influenced by changes in the size of the population and the number and type of variables included. For instance, approximately 7.3% of records within the 1990 census are unique, or potentially identifiable, given the 100,000-person population threshold using standard census variables such as age, race, ethnicity, sex, and housing or household information [23]. Nevertheless, the proportion of unique records is a function of the available information. Sharing a greater number of variables increases the potential to identify an individual; therefore, the Census Bureau population threshold increases from 100,000 to ≥250,000 when greater numbers of variables are released as microdata [20].

There is a point at which increasing the size of the population no longer adds notable increases to data protection. For census data, when only 6 demographic variables are shared, there is a point of diminishing returns for approximately 20,000 people [21]. In addition to the number of demographic variables, the type of variables shared also matters. For instance, a population of 25,000 contains 25% unique records when 9 variables are shared; however, when the occupation variable is removed, this proportion drops to 10% [22]. In this case, occupation can be particularly identified, given that some occupations are much rarer than others. The HHS drew on this scholarship to make their determination [23]:

After evaluating current practices and recognizing the expressed need for some geographic indicators in otherwise de-identified databases, we concluded that permitting geographic identifiers that define populations of greater than 20,000 individuals is an appropriate standard that balances privacy interests against desirable uses of de-identified data. In making this determination, we focused on the studies by the Bureau of Census cited above which seemed to indicate that a population size of 20,000 was an appropriate cut off if there were relatively few (6) demographic variables in the database. Our belief is that, after removing the required identifiers to meet the safe harbor standards, the number of demographic variables retained in the databases will be relatively small, so that it is appropriate to accept a relatively low number as a minimum geographic size.

In addition, as the HHS considers the 20,000-person population stipulation, the lowest bound could also be tied to the adoption of the 3-digit zip. Although 3-digit zip codes vary widely in terms of the size of the population they contain (in 2020, ranging from 3147 to 3,310,455 people), only 18 zip codes of 3 digits containing <20,000 people at the time the safe harbor was first determined. Currently, there are only 13 zip codes of 3 digits in the nation, which are too small and would need to be merged with neighboring geographies to meet the minimum threshold of 20,000 people [24]. Fortunately, as most 3-digit zip codes contain populations of >20,000 people, researchers following the 3-digit zip code rule are not often burdened with the task of data aggregation. Perhaps the HHS hoped that using these 3-digit zip codes could help enforce a more conservative following of the population threshold while also making the guidelines more straightforward. Unfortunately, this is not the case in many important ways.

## Twin Challenge 1: Ambiguity

### Overview

The safe harbor rule seems straightforward when seen from the original final rule of 2000; however, given the modifications, as well as how it appears in the literature today, it carries an essential ambiguity that has led to large gaps and disagreements in research and policy work. We first examine different interpretations of the rule based on these ambiguities and draw examples from the scientific literature to show how different scholars rely on different interpretations. We then simplify the discussion by proposing that the crux of many disagreements—and the basis of productive ways forward—can be seen by focusing on the use of 3-digit and 5-digit zip codes.

### Safe Harbor Provision and Zip Code Ambiguity

The primary driver of disagreements in the literature seems to hinge on how individual researchers and teams interpret the role of zip codes versus the 20,000-person threshold. This often comes to the fore in determining how much location data must be removed from patient data to satisfy HIPAA requirements.

The potential for misunderstanding stems from one part of the provision—the piece regarding geographic information that states the following with respect to patient location data: all geographic subdivisions smaller than a state, including street address, city, county, precinct, zip code, and their equivalent geocodes, except for the initial 3 digits of the zip code if, according to the current publicly available data from the Bureau of the Census: the geographic unit formed by combining all zip codes with the same 3 initial digits contains >20,000 people, and the initial 3 digits of a zip code for all such geographic units containing ≤20,000 people is changed to 000.

An understanding of the HIPAA safe harbor rule has been further muddied by the different ways in which it is described by experts in the fields of public health and geography and by the guidance of the HHS and the OCR. A reader of the *background and context* section on the *2010 De-Identification Standard Workshop* page on the HHS website [11] could justifiably conclude that any aggregation of 20,000 people is in compliance with the safe harbor rule regardless of zip code. In contrast, focusing on the zip code rules as they appear in the literature could lead a person to conclude that zip codes are the primary vehicle for data protection. This is because, in many cases, authors simply do not specify the type of zip code used in their work. This potential for ambiguity among different sources has likely contributed to the number of studies that have aggregated (or suggested the possibility of aggregating) in ways that do not align with the 2000 HIPAA final rule [8,25-27]. Textbox 2 offers a number of different justifications for how scholars have interpreted the safe harbor provisions.

The fact that a range of views exists is not surprising, considering the ways in which HIPAA provisions have been interpreted within the fast-growing scholarly literature using spatial health data and among various web-based help resources. Understanding of the safe harbor provision is muddied by conflicting or ambiguous phrases that appear across a broad array of resources and by how different scholars seem to follow different practices and procedures for handling patient location data. This profusion of differing practices, although perhaps engendering interesting conversations, likely comes at the cost of research output being unnecessarily overly masked to protect sensitive health data.

### Two Different Interpretations

To find a way forward toward more standardized interpretations of HIPAA safe harbor rules, it helps to delineate 2 distinct ways of interpreting the safe harbor provision specific to location data (while recognizing that less common interpretations may also exist). In essence, 2 different and competing interpretations have emerged: the 3-digit zip interpretation and the 5-digit zip interpretation.

#### The 3-Digit Zip Code Interpretation

For many health researchers, there is only one interpretation of the safe harbor provisions. This is likely because the privacy rule was designed with tabular data in mind, and much medical research involves working with data in its tabular form [9]. For these investigators, a zip code is primarily a 5-digit number that can be reduced to a 3-digit one [5]. For example, an analyst receives a spreadsheet of patient data from which to build a risk model. One column in the table would be designated for the location attribute (ie, a column for zip codes). According to this rule, only the first 3 digits of the zip code are permitted to be shared (unless the population value is <20,000, whereby the data are suppressed or converted to 000). For most lawyers, medical researchers, and those using patient data in tabular format, there is little ambiguity in the safe harbor standard.

#### The 5-Digit Zip Code Interpretation

**Figure 1:**
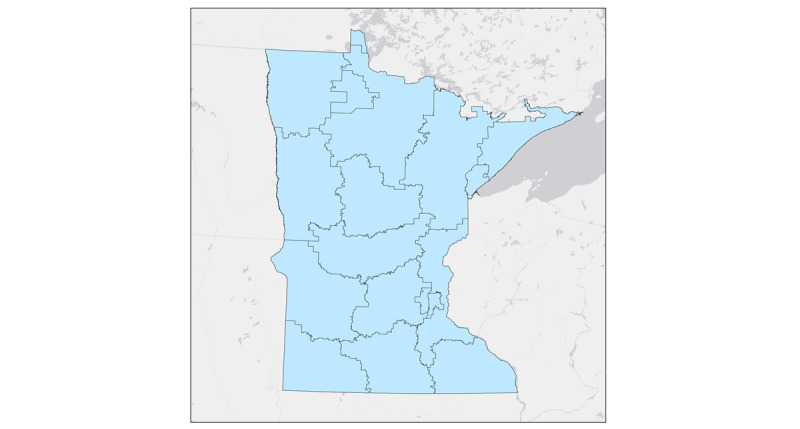
Three-digit zip code boundaries.

**Figure 2:**
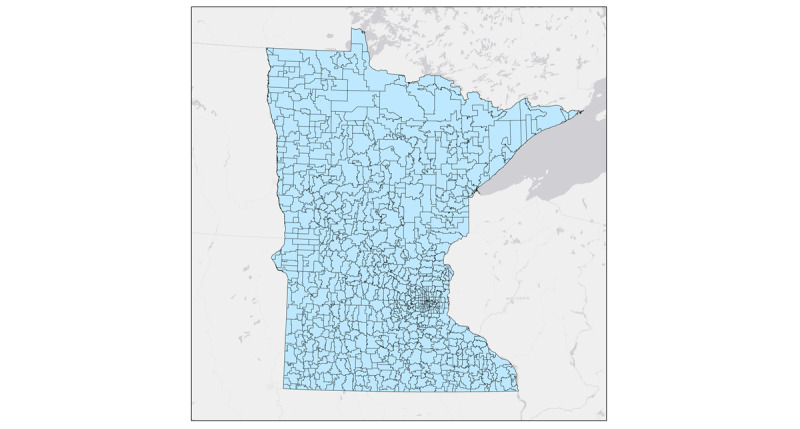
Five-digit zip code boundaries.

**Figure 3:**
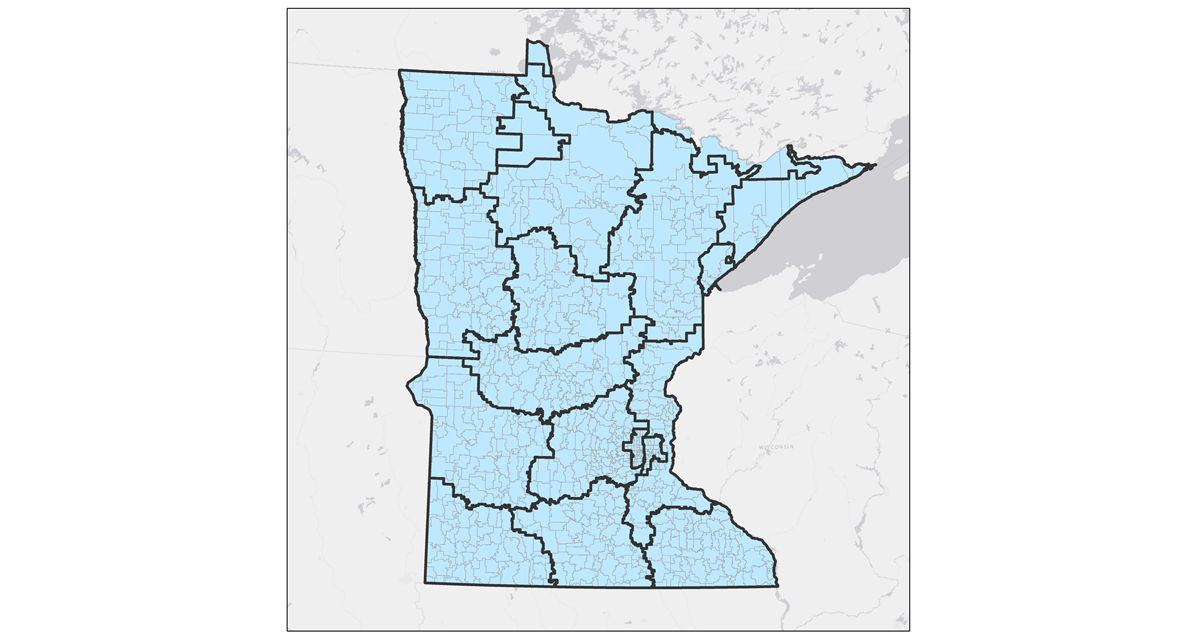
Five-digit zip codes nested within three-digit zip codes.

For those who view zip code data primarily as spatial data, the privacy rule elicits some confusion. Although a zip code is a 5-digit number, to geographers and a growing number of other scholars who use spatial data, it is also an area on a map. Zip codes divide regions into smaller areas designed to aid post delivery. Both 3-digit zip code areas (Figure 1) and 5-digit zip code areas (Figure 2) are present. The 5-digit zip code areas are nested within 3-digit zip code areas (Figure 3). People who work with spatial data are likely to be familiar with this hierarchy of spatially nesting areas and how it can lead to conflicting interpretations of provision §164.514(b)(2a), which states the following:

(2a) The geographic unit formed by combining all zip codes with the same three initial digits contains more than 20,000 people

In this view, there are 2 ways of reading “Zip codes with the same three initial digits,” namely either (1) using 3-digit zip codes (as described in the previous paragraph) or (2) using 5-digit zip codes that share the same 3 initial digits.

**Figure 4:**
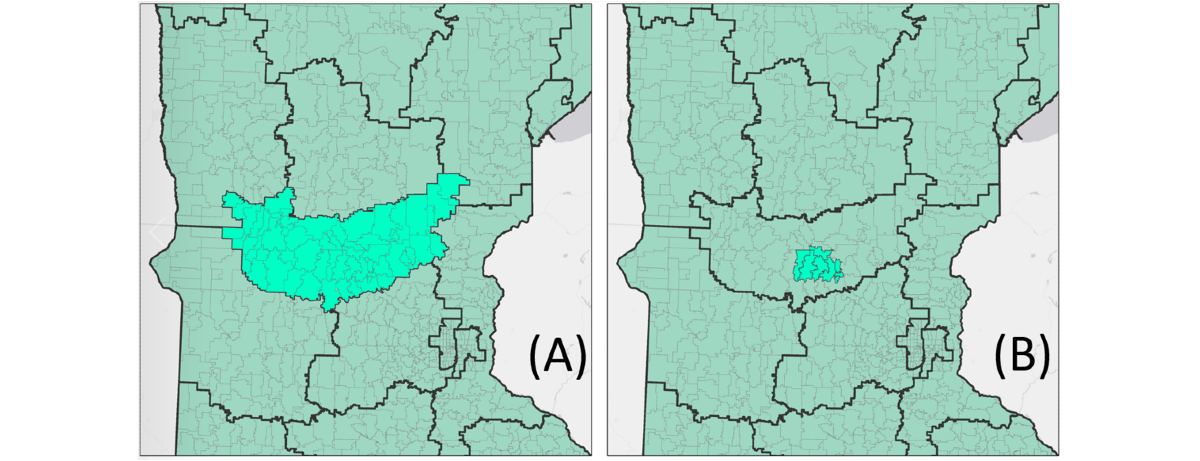
(A) All the 5-digit zip codes beginning in “563.” (B) An aggregation of 5-digit zip codes that all begin with “563” and contain >20,000 people.

The root of this apparent ambiguity comes from the phrase “all zip codes.” If we interpret “all zip codes” as “all of the five-digit zip codes,” then the 3-digit zip code rule would still apply, as when one combines all the 5-digit zip codes together, they are left with a 3-digit zip code area (Figure 4). However, if “all zip codes” were interpreted as “all five-digit zip codes within the aggregation,” a less conservative interpretation emerges where 5-digit zip codes can be combined to meet the 20,000 population threshold as long as all the used 5-digit zip codes have the same 3 initial digits (Figure 4). Simply put, this interpretation permits investigators to aggregate 5-digit zip codes when they all fall within the same 3-digit zip code area. The large difference in the areas highlighted in Figures 1 and 2 demonstrates the impact of these 2 competing interpretations. Here, we must note that the 5-digit interpretation does not meet HIPAA standards; the reasons for this are discussed later in this paper.

### Drivers and Implications of the 2 Interpretations

**Figure 5:**
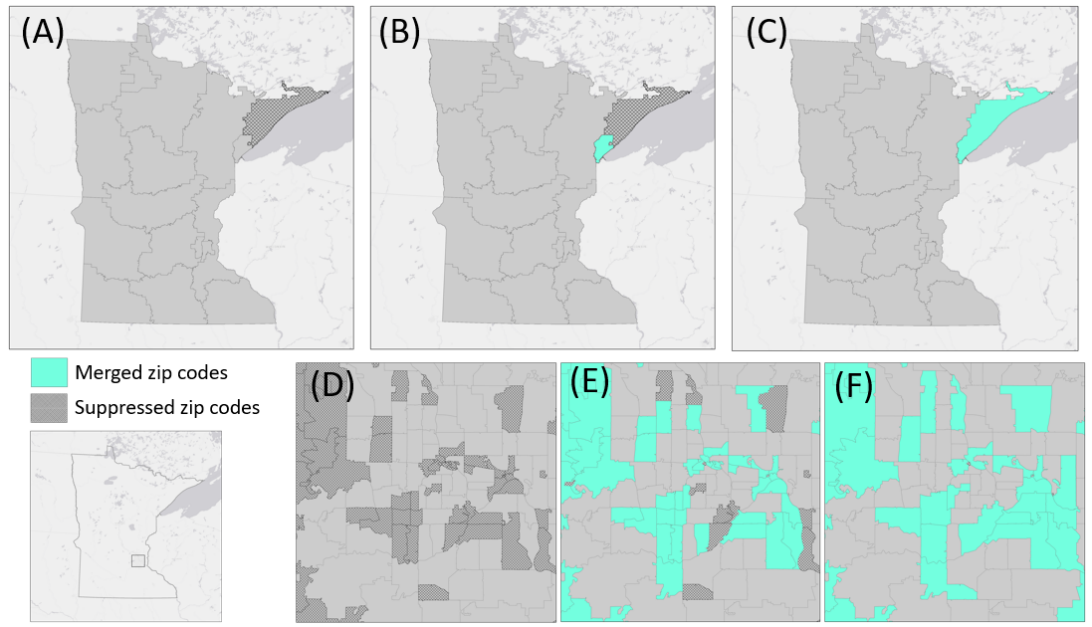
The aggregation process as seen within (A-C) 3-digit zip codes (D-F) and 5-digit zip codes. Zip codes with populations <20,000 people are suppressed. To address suppression, low-population zip codes are merged with neighboring zip codes to meet Health Insurance Portability and Accountability Act requirements. It is not in adherence with Health Insurance Portability and Accountability Act Safe Harbor to use 5-digit zip codes as the unit of aggregation.

Comparing studies that use 3-digit versus 5-digit zip codes illuminates a potential cause for the existence of competing interpretations tied to whether the work uses tabular data or spatial data. In the case of either 3- or 5-digit zip code interpretation, tabular data can appear in essentially the same format (containing only the first 3 digits of a zip code). However, the same mapped data would be *very* different. A researcher operating under the 3-digit interpretation would share maps of patient data at the 3-digit zip code level (Figure 5), and if a 3-digit zip code contained <20,000 people, it would be merged with a neighboring unit. The corresponding tabular data for these maps would only contain 3-digit zip codes. However, investigators operating under the 5-digit zip code interpretation could share maps at the 5-digit zip code level; if the 5-digit zip code contained <20,000 people, it would be merged with neighboring units that share the same first initial digits. The corresponding tabular data for these maps would only contain the first 3 digits of a zip code as well; however, as >1 aggregation would fall within each 3-digit zip code area, there would be multiple records with the same 3-digit zip code.

These differences are not hypothetical as relevant examples are abundant in the literature. Bearing in mind that researchers rarely describe their decision-making in detail, there is a body of work that seems to operate under the 3-digit zip code interpretation [8,10,17,27-30]. Another realm of scholarship appears to operate under the 5-digit zip code interpretation [4,26,30], and there is related work that seems to suggest the capability of aggregating any geocode to meet the 20,000 threshold [7,8,25]. These are some of the many potential examples of how there appears to be a divide between the 3- and 5-digit zip code interpretations of HIPAA.

Interestingly, there appears to be some commonality within and differences among disciplines regarding the way a safe harbor is interpreted. Although this paper does not attempt to conduct a full literature review, anecdotally, of the studies cited in the previous paragraph, all those operating under the 3-digit zip code interpretation are authored by epidemiologists, medical researchers, or computer and information scientists, whereas the papers backing the 5-digit zip code interpretation are authored by geographers. Although this is just a sample of a larger literature, there seems to be a trend where spatially oriented researchers are more likely to embrace the 5-digit interpretation or a more lenient understanding of the rules around a threshold of 20,000 people. This is not surprising, given that geographic research often necessitates a map, and 3-digit zip codes are not intuitive map units. It is also the case that 3-digit zip codes are not easy to find in the form of public shapefiles, or mapping files, that are often used for research. Neither Census [31] nor the US Geological Survey offers data at the 3-digit zip code level. In fact, at the time of writing, we can only find 2 sources that provide data for download in the form of 3-digit zip code boundaries for the United States, and both sources are proprietary (Esri’s ArcGIS Online and Caliper’s Maptitude). Even without access to these proprietary resources, it is possible to create boundaries on one’s own. However, one would think that as 3-digit zip codes are the required units for display under HIPAA law, they should be more readily available on the web. In contrast, data at the 5-digit zip code level are easy to find on the web and appear abundantly in the public health literature. The extent to which the dearth of 3-digit zip code map data plays a role in the misunderstanding of the safe harbor rule is unclear; however, one cannot help but wonder whether the widespread confusion would exist if 3-digit zip code mapping files were available for download on the HHS website.

The potential implications of misunderstanding privacy guidelines are profound when considering that researchers share patient data in inconsistent ways that bear on both the efficacy of health interventions and the potential for privacy breaches. When studies share aggregated patient data at the 3-digit zip code level, their output is generally not useful for identifying local distributions of health and disease, although they provide a more generous degree of data security. When studies share PHI at the 5-digit zip code level, they can provide a much more useful depiction of the spatial health dynamics at hand but at the cost of weaker data privacy.

In terms of this trade-off, the difference in identification risk between 3-digit and 5-digit zip codes is substantial enough to warrant an alarm, as discussed in detail in the following section [15]. At the same time, the difference in spatial resolution between the 2 forms of zip codes carries potentially problematic costs. For instance, one study demonstrated how different disease patterns emerge depending on whether 3-digit or 5-digit zip code areas are used, and with an example data set, the authors showed that if 3-digit zip code areas are used to determine how to best distribute N95 respirators during a pandemic, it would result in a surplus of supplies for health care workers in some communities and shortages others [30].

## Twin Challenge 2: Data Loss

### Overview

Even after gaining a clearer understanding of HIPAA law and how it is meant to be interpreted, one more challenge remains, namely that HIPAA guidelines are very likely too strict in general, resulting in an unnecessarily large degree of data loss [3,17]. The following sections provide insight into the extent of the data loss that occurs when adhering to HIPAA Safe Harbor’s 3-digit zip code rule and how other (non-HIPAA–compliant) interpretations can reduce data loss without adding much in terms of privacy risk, depending on the types and amount of data being shared.

### Data Loss From 3-Digit Zip Codes and 20,000 People

**Figure 6:**
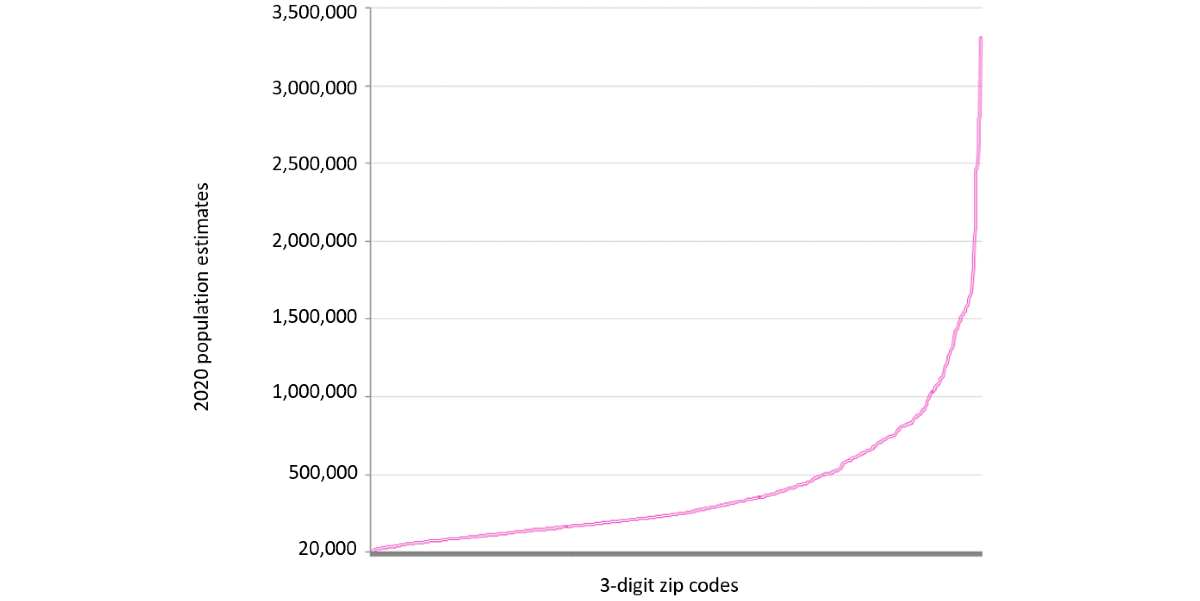
Three-digit zip codes (100-999) ordered least to greatest by population from 2020 estimates from the American Community Survey.

Opting for the 3-digit zip code interpretation is a conservative choice that has a number of negative implications for research and policy. The 3-digit zip code interpretation is very cautious with respect to adhering to the 20,000-person rule. Bear in mind that, as of 2020, the average population contained within a 3-digit zip code is 397,372 people, which is almost 4 times the population threshold of 100,000 required by the Bureau of the Census for the release of microdata (individual response data from the census). Thirty years after the initial rule, there are now only 13 zip codes of 3 digits that require suppression (as they have <20,000 people in them). The number of ideal units containing small but acceptable populations is disappointingly low; only 12 units contain between 20,000 and 30,000 people, and only 21 contain between 30,000 and 40,000 people. Just over 91% of 3-digit zip code geographies contain >60,000 people or at least 3 times the 20,000-person threshold. In simple terms, we should expect that most geographies shared under the 3-digit zip code safe harbor standard will contain populations far greater than the 20,000-person threshold (Figure 6).

Given that most 3-digit zip code geographies contain >20,000 people, under the HIPAA safe harbor provision, most will have a very small proportion of unique records. However, some places will have a proportion of unique records that are considered relatively riskier in terms of patient protection. In any case, the small number of instances that contain the “riskier” low-level minimum populations still meet the minimum acceptable level of risk (which, if we look back at the simulation study by Horm [22], would result in approximately 10% unique records). This is slightly higher than the 7.3% estimated unique records in the 1990 census microdata; however, the HHS points out that the actual risk will be much lower because of the limited number of publicly available tables that can be used to compare the patient data with. These risk estimates are also subject to the myth of the perfect population register, which is discussed later in this paper [17]. Finally, the HHS suggests that the relatively low probability of success should be a deterrent in and of itself.

An interpretation of this threshold is that if the HHS is satisfied with some units being shared at the level of 20,000 people, could all units be shared at that resolution? After all, if populations of 20,000 meet the minimum acceptable level of risk, then what is stopping investigators from aggregating 5-digit zip codes to meet this requirement? Zip codes of 3 digits are rather impractical for research purposes; hence, it is very uncommon to find a map shared at this level. For this reason, it is easy to see how researchers could come to believe that the 5-digit interpretation is permissible if they have not given the legal documents a thorough reading.

Aggregating 5-digit zip codes to create the finest-grained units possible that also still meet the 20,000-person threshold is tempting as this would allow investigators to meet the minimum acceptable level of risk in a way that enables the sharing of maps with more detailed and consistent geographies than that provided by 3-digit zip codes. In this scenario, there would be a slightly greater risk of identification because of the minimum population size, although it would still seem to be an acceptable level of risk as long as the 18 other safe harbor–restricted identifiers were removed. The remaining problem is that 1 of the 18 identifiers is not being *fully* removed in this scenario. By aggregating 5-digit zip codes, an individual record contains more information than a single 3-digit zip code; in addition, it now contains a handful of 5-digit zip codes that can be used to further narrow down the possible matches. Therefore, *5-digit zip code aggregations do not meet HIPAA safe harbor standards*.

However, depending on what other information is kept, it is reasonable to believe that sharing a map of patient data stripped of age and other demographics at the aggregated 5-digit zip code level would lead to a very low (certainly quite low) risk of identification. One study showed that certain elements from a list of 18 identifiers can still be shared without jeopardizing patient privacy “when other features are reduced in granularity.” Specifically, Malin et al [28] found that more detailed age data (beyond what is permitted by safe harbors) could be shared when they coarsened the specificity of other variables such as ethnicity [28]. The authors noted that every data set is different, and because of this, alternative deidentification practices can be used to enable the safe disclosure of patient data that are normally suppressed under the safe harbor method. This means that there is potential for 5-digit zip code information to be safely shared in an aggregated form as long as other identifying information is suppressed.

In summary, it may be time to rethink the one-size-fits-all strategy, which is the safe harbor method. It is reasonable to ask whether aggregating 5-digit zip codes into regions that contain at least 20,000 people could achieve a “sufficiently low” risk of identification when other patient information is suppressed, such as date of birth (DoB) and gender. It would be even more reasonable to suggest that aggregating 5-digit zip codes could work if no patient information other than diagnosis and location was shared. Curtis et al [6] tested this claim in a study that found that when put to the test, students were unable to identify individuals in simulated cancer maps. There was little reengineering risk, even at aggregated resolutions of finer than 20,000 people. To this point, this paper has pointed out the ambiguities within the safe harbor standard while shedding light on some of the arbitrary determinations made by the HHS that have contributed to a perhaps overly conservative definition of privacy. The following section takes a closer look at how the safe harbor rule has been criticized for being too stringent and, at the same time, not sufficiently protective, specifically when it comes to identification risk.

### Do the Privacy Gains Justify the Amount of Data Loss?

To dive deeper, we must go back and consider the influence of the population-level identification attack by Sweeney [15]. As stated previously, this initially resulted in the decision to bar both 3-digit and 5-digit zip codes from deidentified data; however, after taking public comments, the HHS reconsidered, and 3-digit zip codes were deemed permissible as long as they contained a population of at least 20,000 people. The HHS justified their restrictions by citing particular studies that led them to believe that the combination of 5-digit zip code, gender, and DoB would be enough to potentially identify a great deal (more than half) of the US population based on uniqueness [32]. Note that to be considered “unique,” a record must contain a combination of characteristics that make it different from all other records in that table [33]. If the number of unique individuals within the US population was as large as Sweeney [15] reported, the motion to block the 5-digit zip code and DoB under safe harbor seems quite justified. However, some have pointed out that the combination of these 3 identifiers, even with their formidable discernibility capabilities, might not be as threatening as the article by Sweeney [15] makes it out to be.

Barth-Jones [17] describes the “myth of the perfect population register” in his 2012 paper, which points out how many investigators often forget to account for the people missing from the lists used to link individuals to their medical records. These missing populations add significant uncertainty to the calculation of true population uniqueness [17]. Therefore, the actual proportion of unique individuals on a list cannot be determined with 100% certainty if potential matches exist off the list. Therefore, these kinds of studies must be careful in the statements they make—oftentimes including phrases such as “likely unique” or “potentially identifying” as certain identification cannot be claimed without a list of the entire population or the knowledge that the individual under identification attack was indeed contained within both lists.

For instance, consider the paper by Sweeney [15], which the 1999 NPRM cites saying “A 1997 MIT study showed that because of the public availability of the Cambridge, Massachusetts voting list, 97 percent of the individuals in Cambridge whose data appeared in a data base which contained only their nine-digit zip code and DoB could be identified with certainty.” [16] According to this, nearly all Cambridge voters can be identified using the combination of DoB and 9-digit zip code. Sweeney [15] states that this proportion of people can be “uniquely identified” on this basis; however, these individuals are only uniquely identifiable within the population of registered voters and not within the general Cambridge population (see the study by Barth-Jones [17] for a full explanation). This means that, for an intruder to identify an individual’s medical record, they must know that the individual exists on both lists and that no other person in Cambridge shares the same DoB and 9-digit zip code. When deciphering the data, the intruder must account for 35,000 nonregistered voting-aged people living in the city, any of whom could be the true subject of the medical record of interest. Unaccounted populations inject much uncertainty into the identification of unique records (35% error in the study by Sweeney [15]). With an imperfect population register, as exemplified by the Cambridge attack, an intruder would be able to identify no one with 100% certainty. Barth-Jones [17] concludes that the governor was likely only identifiable based on the fact that he was a public figure who had public hospitalization. The date of hospitalization was known, as well as his DoB, gender, and zip code; moreover, it could be easily assumed that he would be a registered voter. In instances such as this (having information a priori), an intruder can be confident of a unique match.

It is unclear whether the HHS wrote the NPRM with a full understanding of the methodological limitations of voter list-based identity attacks of the kind described by Barth-Jones [17]. It is possible that the clause “...could be identified with certainty” was taken without really considering the implications of the prior clause “...whose data appeared in the data base.” Many assumptions must be met before we can ignore the myth of the perfect population register. In this example, to identify 97% of the individuals with certainty, we would need to be sure that none of the 54,805 voters on the voter list had the same birth date as a nonvoter living in their neighborhood. We might then wonder how 97% could be identified on the list compared with the proportion identifiable in the entire Cambridge population. This is something we cannot determine as we do not have a population register. However, given that the total population of Cambridge is approximately 88,000 [17], there is much room for error. If the HHS based its development of safe harbors on a limited understanding of these complexities, it might lead us to wonder whether the level of protection delineated within the safe harbor standard is overly conservative.

Nevertheless, even if the HHS misunderstood how Sweeney [15] was using the term “identifiable” in her 1997 paper, there is still room for concern about how far to read into the study. The work by Sweeney [15] is bold, insightful, and conveys a critical message: private information is vulnerable to attacks. The extent to which we *understand* the vulnerability is unclear. Even with the injection of uncertainty from missing populations, the risk for identification may still be considered too high and the implications would be quite serious. Let us return to the Barth-Jones [17] review of the attack by Sweeney [15], which finds that somewhat fewer (but perhaps not much fewer) than 29,000 people out of 88,000 in Cambridge are identifiable (if the record is unique and the data intruder already knows that the individual is on both lists). Depending on the motive of the data intruder, this might not be far from likely. It is easier to link a specific person to their medical record than to link a specific medical record to the person to which it belongs. This is because a motivated attacker is likely to have collected background information on the person a priori. The data intruder likely has a target in mind—someone they know—and therefore, it is not that unlikely for them to already have information on the target’s voting behaviors and place of work, allowing the intruder to determine the employment insurance coverage that could be used to confirm the target’s presence on the insurance hospitalization data list. Moreover, even without knowing with certainty if the target of the attack is on both lists, the fact that the chance of a false positive (matching a record to a voter on the list when the record actually belongs to a nonregistered voter) occurring could be perceived as highly unlikely by the attacker, which could encourage them to continue with their plans regardless of the potential false positive.

The combination of DoB, gender, and 5-digit zip codes can be problematic when shared in conjunction. The question that remains is whether this combination of identifiers can be reworked to reduce the risk of identification. In the literature on microdata anonymity, zip code, gender, and DoB are actually not considered full identifiers themselves but rather quasi-identifiers that can be used in combination to find unique instances. The term “identifier” is reserved for information that uniquely identifies an individual, such as a social security number [34]. Nevertheless, quasi-identifiers can be dangerous when used in combination; however, how dangerous are they? To gain some insight into this question, we must look more closely at how identification risk has appeared in the literature, relying on the HIPAA safe harbor method.

### What Level of Data Loss Defines Sufficient Data Protection?

What is the acceptable level of identification risk? There is no universally recognized standard that defines what a sufficient proportion of unique records should be. Some have suggested that the nationally accepted standard of reidentification risk is defined by HIPAA’s safe harbor standard itself [27] but recall that the safe harbor standard was derived somewhat arbitrarily, being loosely based on rules used by the Bureau of the Census and a couple of simulation studies. In fact, when determining the population requirement of the HIPAA safe harbor rule, the HHS made the following statement in regard to defining “minimal risk”:

With respect to how we might clarify the requirement to achieve a “low probability” that information could be identified, the Statistical Policy Working Paper 22 referenced [see 18 in our references] discusses the attempts of several researchers to define mathematical measures of disclosure risk only to conclude that “more research into defining a computable measure of risk is necessary.” When we considered whether we could specify a maximum level of risk of disclosure with some precision (such as a probability or risk of identification of <0.01), we concluded that it is premature to assign mathematical precision to the “art” of deidentification.

Twenty years later, there is still no threshold defining “sufficiently low probability,” and investigators fall back on the safe harbor standard as a point of reference for comparing different levels of data protection. Deidentification with the safe harbor method is said to leave somewhere around 0.03% or 0.04% of records within the US population vulnerable to identification [17,35]; however, this proportion fluctuates according to the geographical extent of the data set, where some regions have much smaller proportions of unique records and others have much higher. Specifically, the reidentification risk has been found to range from 0.01% to 0.19% [28], 0.01% to 0.25% [36], and 0.013% to 0.22% [37] on a state-by-state basis.

Most studies estimate the identification risk under a safe harbor to be low. However, there is no consensus on whether safe harbor standards are sufficient to protect patient data. In other words, “sufficiently de-identified” is subjective and, on occasion, very similar proportions of unique records have evoked very different assessments. For example, Sweeney asserts that the estimated safe harbor reidentification risk of 0.04% of the US population is not a sufficient privacy guard [10,35], whereas Barth-Jones [17] suggested that the risk would actually be <0.03% (when using a voter list attack strategy) and that this proportion is, in fact, sufficient; he goes on to compare the identification risk under a safe harbor to the likelihood of being struck by lightning [17]. A reidentification attack by Kwok et al [37] reidentified only 2 of 15,000 individuals (0.013%) from a safe harbor protected data set, and the intruder was provided with a substantial amount of information from a market research company. Kwok et al [37] concluded that there was a low risk of reidentification and that masking with a safe harbor makes reidentification a challenging task. Others asserted that the safe harbor is too stringent. Malin et al [28] suggested in a 2011 article that the safe harbor method was too conservative as it is possible to release more detailed information without presenting a greater risk than that provided by the safe harbor method. In contrast, a 2016 study found that even when data seem sufficiently masked, computer science models can be used to identify a large proportion (42.8%) of patients by linking demographics such as age, sex, hospital, and year [38]. Although specific to a single case study, this is a high and likely unacceptable level of risk. More recently, Janmey and Elkin [27] suggested that the safe harbor standard is sufficient for preserving privacy at an overall population level. However, they also found that encounter notes within data can sometimes include indirect identifiers that can be used to help match records, and this could increase the risk of identification to 0.07%, which is well over the estimated range of risk previously mentioned when using safe harbor [17,35].

It is safe to say that there is disagreement regarding what is sufficient for data protection. This type of risk calculation is complicated in and of itself and a concept such as *sufficiency* is necessarily a judgment call. Identification risk depends not only on how the data are released but also on the alternative lists publicly available to the data intruder. Sweeney [10] described how identification risk for safe harbor–abiding data sets can be as high as 25% when the intruder uses more than just a voter registration list. Other detailed registries can be used to reidentify masked data such as real estate tax data, credit reports, and property records. Moreover, identification risk can foreseeably jump much higher—far beyond the expected ranges—for certain areas where the demographics of the base populations allow an intruder to easily narrow down potential matches based on age or ethnicity, as seen in regions dominated by college dorms, ethnic enclaves, or transient communities [15,38]. Sufficient data protection (leaving aside the definition of sufficiency) will always be dependent on the data set being masked as a slew of factors determines the overall identification risk.

## Ways Forward

### Overview

So far, we have focused on 2 key issues of safe harbor provisions: the confusion around which zip codes to use and whether the rule warrants an unnecessarily large amount of data loss. Reviewing the process by which the safe harbor concept came into being provides insight into the intended interpretation of the provision and the motivations that guided its development; however, this is the first step. The ambiguity of how to best interpret and use zip codes or other geographic identifiers persists, and there is no clear consensus on what defines sufficient minimal risk. In this paper, we explore new approaches to data privacy and how they may meet the needs of some researchers; however, we conclude by arguing that the most promising way forward to addressing the twin problems of safe harbor is to steer away from one-size-fits-all guidelines and toward deeper assessments of domain-specific and data-specific modes of masking that could offer a middle ground between useful data and protected data.

### New Approaches to Deidentification

In the face of the complex nature of reidentification risk, scholars and policy makers have begun to advocate for the widespread adoption of k-anonymity or differential privacy (DP) methods [10]. The primary argument for these approaches is that deidentification methods should come with privacy guarantees, especially as technology advances and powerful automated systems can be made to search for matches between multiple public lists. Therefore, although k-anonymity and DP cannot necessarily guarantee data security, these methods have been receiving considerable attention recently as they provide a type of privacy guarantee that offers more complete data protection than traditional masking approaches.

K-anonymity ensures that no unique records exist in the data set and further requires that each record has a minimum of “k-1” common records (those that have the same quasi-identifiers) so that they cannot be differentiated and therefore identified with certainty [39]. K-anonymity can be achieved through many traditional methods such as jittering, aggregation, and location swapping, and it often provides a higher level of protection than if one were to use one of these traditional methods alone. However, k-anonymity is not impervious to intruder attacks. An intruder can still use background knowledge to narrow down the possible matches to increase the likelihood of identification, such as in a homogeneity attack (attacks based on data that contain identical values for an attribute), in which a region with a homogeneous population containing similar values for a record in the table can be used (alone or linked with other data) to identify an individual or diagnosis. Therefore, k-anonymity, strictly speaking, does not guarantee privacy. However, it guarantees nonuniqueness, which, in the absence of outside knowledge, provides considerable data protection, and therefore, k-anonymity remains a popular approach.

DP is attracting attention as a newer approach to protecting sensitive data that assures a very low likelihood of individual identification. The most common definition of DP is that of epsilon DP introduced by Dwork et al [40]. The epsilon DP by Dwork et al [40] involves creating a synthetic aggregated data set from an original unprotected data set, which ensures that an individual record cannot be identified. These simulated data are built by injecting a predetermined amount of noise (based on a Laplace distribution) into the original aggregate table such that it does not significantly influence the output (of queries into particular prespecified relationships). In other words, the aggregate table is systematically adjusted to secure individual privacy while also ensuring that the data provide similar results to what would have been given if the original data were used in a prespecified analytical model. This is achieved such that if any one individual was removed from the data set, it would not influence the overall results. This means that epsilon DP provides relative guarantees about disclosure risk, and essentially promises that “...any given disclosure will be, within a small multiplicative factor, just as likely whether or not the individual participates in the database.” [40]

Unlike k-anonymity, DP protects data under the assumption that an intruder has close to perfect knowledge, and in doing so, DP offers a level of protection unlike others. DP does not succumb to the same weaknesses as traditional methods (including the homogeneity attack) and provides stronger data protection against differencing, linkage, and reconstruction attacks [41]. In addition, because of its robustness, DP has the advantage of reducing improper data analysis techniques by limiting the ability of a single observation to have an effect on the result, which helps to deter things such as p-hacking, hypothesizing after the results are known, and overfitting models [42]. For these, and many other reasons, DP has gained considerable attention over the past 2 decades. In fact, DP methods have the potential to replace existing masking methods and have already been adopted by Apple and the Bureau of the Census, which intends to use DP to protect the 2020 census microdata. DP is not infallible; it offers “an extremely strong guarantee, it does not promise unconditional freedom from harm.” [41]

As DP provides a higher level of protection than many other methods, it potentially offers a way for researchers to share data at more detailed levels than previously allowed in safe harbors. In an example of disease surveillance mapping, the safe harbors’ minimum population requirement of 20,000 people is rather limited in terms of map resolution. A map with units containing 20,000 people would not provide enough detail to be helpful to researchers, policymakers, or community members. However, DP would allow investigators to share maps at much finer scales (down to the neighborhood level) without putting patient identities at risk.

Thus, why not use DP? This is because it has critical drawbacks for research use [43]. For instance, a map created from a differentially private aggregated table displays simulated data; therefore, it is possible that some regions on the map would not accurately reflect the original data, especially at finer scales where the population numbers are lower. Santos-Lozada et al [44] found that the infusion of noise from DP methods affects observed distributions differently for different demographics, meaning that DP has the potential to bias the understanding of health disparities at the national level. In particular, the authors demonstrated how mapping differentially private data led to “overestimates of population-level health metrics of minority populations in smaller areas and underestimates of mortality levels in more populated ones,” and these effects were dramatic. For instance, note the following:

...in McCulloch County, Texas, the mortality rate ratio for non-Hispanic blacks is 75.9, indicating the mortality rate would be 24% lower under the current methodology compared with the differential privacy methodology. Similarly, in Clarke County, Virginia, the mortality rate ratio for Hispanics is 121.4, indicating the mortality rate would be 21% higher under the current methodology compared with the differential privacy methodology. At the same time, the non-Hispanic white mortality rate ratios were essentially unchanged for these two counties, at 100.3 and 99.8, respectively, meaning substantial biases may enter into understandings of disparities.

The implications of DP for research are dire, and the recent move by the Bureau of the Census to adopt this approach for the 2020 census microdata has drawn much attention to its advantages and disadvantages [45,46]. Census data are one of the largest sources of sociodemographic data used by social scientists; therefore, differentially private methods threaten to degrade the reliability and effectiveness of social science research. Other than threats to data accuracy and biases, another source of concern regarding the 2020 census data is that these differentially private tables would not enable exploratory data analysis. This is because differentially private data are synthetic, and therefore, relationships cannot be explored unless they are prespecified when the synthetic table is created. For this, it is very likely for DP to interfere with the process of data-driven scientific research, pushing some scholars to suggest that perhaps “...differential privacy goes far beyond what is necessary to keep data safe” [46].

There is much uncertainty regarding the practicality of DP for the protection of large-scale, sensitive data. DP is a relatively new concept for several social scientists and epidemiologists. There is a dearth of investigations into DP within the social science literature, particularly regarding the impact it might have on health mapping. We could only find 1 study at the time of writing [44] but expected more, given the attention paid to DP and the many unanswered questions that it poses. What are the implications of DP in mapping in terms of accuracy and use? How do differentially private maps compare with maps of the original raw data? Furthermore, it is unclear how DP stands within institutional review boards. This is relatively new territory, and it is likely that many HIPAA compliance officers are unfamiliar with DP. As part of our examination of the history of HIPAA, we spoke with legal experts and HIPAA compliance officers. One such officer, on being introduced to DP, stated that “this doesn’t play into our office’s considerations of deidentification.” DP holds some promise for mapping spatial data but at known and unknown costs.

### Current State and Future Research

Despite the ongoing interest in expanding the use and sharing of health data mapping, the safe harbor rule stands as the primary guidance for those interested in sharing maps. It is far from perfect in that for many scholars, it is ambiguous and either too stringent or insufficient in terms of securing data or reducing data loss. Alternative methods exist, which have the potential to do a better job; however, they have their own drawbacks. HIPAA safe harbor provisions do not set out to guarantee data protection similar to the newer modes of data protection; instead, they only ensure a low risk of identification with the ultimate goal being “to balance the needs of the individual with the needs of the society” [18]. The challenge is to find the “sweet spot” between protected data and useful data while also understanding that this sweet spot changes for each data set depending on what and how much information is available to the public. Furthermore, with rapidly evolving technologies, this sweet spot will continue to change over time. The amount of individual-level data collected by companies today is large and continuously growing. In fact, society may have already reached the point where the myth of the perfect population register is no longer a myth in the face of big data [47].

Although safe harbor continues to stand as the primary source of guidance for handling spatial health data, researchers continue to work with and against it in ways that reflect their understanding of the law and their data against a larger sociotechnical backdrop. As demonstrated by Malin et al [28], there are ways of safely sharing more detailed data (ie, age information) by coarsening the granularity of other data. From this example, we can assume that there are also ways of sharing fine-grained geographic data by censoring other elements in the data. Given that some pieces of information contribute more heavily to individual identification than others (ie, DoB being more identifying than gender), we are left to ask questions that, if answered, could help inform future approaches. Could a 5-digit zip code become innocuous without age information? How many individuals can be uniquely identified by age and 5-digit zip code alone? What if all age and gender information were removed? Would a 5-digit zip code still have the power to identify an individual? In other words, is it reckless to share maps at the 5-digit zip code level if all other patient information is removed (ie, only the sharing of the 5-digit zip code and diagnosis)? What if these zip codes were aggregated to form units that each contained 20,000 people within them? What would be the risk for identification? Of course, it is easier to ask these questions than answer them; however, by examining the history of HIPAA and clarifying the importance of 3-digit zip codes versus 5-digit zip codes, we have a stronger foundation for answering these questions. Until then, the safe harbor method stands as our primary mode of guidance, and 2 decades after its introduction, these guidelines do not meet the public’s need for data security or researchers’ need for useful data.

### Conclusions

Vague privacy provisions stand as an obstacle to progress and pose a threat to public privacy by hindering the ways in which epidemiologists and geographers understand how to share spatial data. This paper promotes an understanding of the HIPAA safe harbor provision by providing a comprehensive overview of the law while also presenting various expert perspectives and relevant studies that, taken together, show how alternative methods to safe harbor can offer researchers better data and better data protection. Two different interpretations of the safe harbor rule exist—the 3-digit and 5-digit zip code interpretation—and although 5-digit zip codes are not the intended level of aggregation under the rule, there is reason to believe that information can be safely shared on a map at this level. More research is needed to determine whether the risk for individual identification is sufficiently low for maps shared at the 5-digit zip code level when DoB and gender are suppressed from the map’s corresponding table. Much has changed in the 20 years since the introduction of the safe harbor provision; however, it continues to be the primary source of guidance (and frustration) for researchers trying to share maps, leaving many waiting for these rules to be revised in accordance with the times.

## Abbreviations

DoB: date of birth
DP: differential privacy
HHS: Department of Health and Human Services
HIPAA: Health Insurance Portability and Accountability Act
NPRM: Notice of Proposed Rulemaking
OCR: Office of Civil Rights
PHI: protected health information

## Availability of Data and Material

Data sharing is not applicable to this paper as no data sets were generated or analyzed during the study.
